# Temporary Cecostomy in Resection of the Distal Portion of the Colon

**Published:** 1918-09

**Authors:** 


***Temporary Cecostomy in Resection of the Distal Portion of the Colon for Non-Obstructive Conditions.*** By Major Gordon Taylor, R. A. M. C. (T. C.). *The British Medical Journal*, June 15, 1918.

The author described what he terms " a little surgical maneuvre " in the resection of the distal half of the large intestine (between the splenic flexure and the sigmoid colon). He advocates temporary or provisional cecostomy in cases of excision of a carcinotomous splenic flexure, descending, iliac, or pelvic colon, where no obstruction exists. The opening acts as vent for gases and prevents strain on the junction.

The author has also used cecostomy to close a proximal inguinal colostomy performed for gunshot wounds. He has usually performed an intraperitoneal operation, excising the " spur " and practising end-to-end union of the bowel. In the excision of a carcinoma and similar operations, he has found temporary cecostomy to be a measure of safety. A stoma is substituted in the right iliac fossa for one of the left side, but, if the cecum is simply anchored to the parietal peritoneum the opening closes readily. The drainage tube is inserted in the manner of a Senn's gastrostomy.

Excision of portions of the distal colon for gunshot injury is desirable in some instances, in spite of the general rule of abstention from operation for wounds of the large intestine, especially when there is infarcation of the bowel. Such a procedure seems preferable to the formation of an artificial anus in cases where resort to this operation is indicated. Excision has usually been performed in cases of gunshot wounds in the distal portion of the large intestine. Provisional cecostomy at the time of resection has proved to be a measure of safety. The " gridiron " method affords convenient access to the cecum. The drainage tube is inserted like a Senn's gastrostomy.

The author makes it clear that he does not advocate excision as an alternative to suture but as an expedient to which it is sometimes justifiable to have recourse. He attributes the idea of operating as described above to Sir Harold Stiles.

